# Hospital Meetings

**Published:** 1904-06-25

**Authors:** 


### HOSPITAL MEETINGS.

### ALEXANDRA HOSPITAL FOR CHILDREN.

The thirty-seventh annual meeting of the Governors and subscribers of this hospital took place on Thursday, 16th inst., Mr. W. H. Whitfield, Chairman, presiding.

In moving the adoption of the report and financial statement, the Chairman drew attention to the signs of progress. The number of beds now available was 97, of which 20 were provided at the Convalescent Home opened at Clandon in July, 1903. The number of children under treatment during the year was 169, and there were 333 out-patients. The 2,150 attendances included visits paid by the out-patient nurse to patients in their own homes. The income from subscriptions was £1,485 12s. 9d., as against £1,387 6s. 4d. in the previous year, while donations amounted to £918, as compared with £775. Two new special cots, supported by annual subscription, had been founded, the donors being Miss Hylda Wertheimer and Mrs. Francis Ball. Miss Jane Arden had endowed, during her lifetime, a cot dedicated to "King Edward VII. and Queen Alexandra on their first Christmas Day after their Coronation as King and Queen." Although, owing to the support extended to the hospital during the year, there was a small balance on the right side the Committee earnestly appealed for additional subscriptions of £1,000, and a special appeal to this effect had been in circulation, and was incorporated with the report. This was rendered necessary on account of the largely increased expenditure, occasioned by the increase in the number of beds at the disposal of the charity, and in consequence of the appeal the Countess of Suffolk and Berkshire had, on her own initiative, organised a scheme which, if carried out, would enable the hospital to continue its work to its full capacity, without constantly appealing to the public.

The report and accounts having been adopted, the re-election of officers took place, the hon. chaplain, the Rev. E. C. Bedford, taking a vacant place on the Committee.

In reply to a question put by Mr. Bedford, as to the possibility of some systematic instruction, by a trained teacher, somewhat on the lines adopted at the invalid schools, being given in the wards, the Chairman said careful consideration should be given to the matter.

Appreciative votes of thanks to the visiting ladies, the medical and surgical staff, and all connected with the work of the hospital having been passed, the proceedings terminated.

### WINIFRED HOUSE INVALID CHILDREN'S CONVALESCENT NURSING HOME, TOLLINGTON PARK, N.

THE annual meeting of Winifred House Invalid Children's Convalescent Nursing Home (Mrs. Hampson's Memorial Home), Tollington Park, N., took place on Monday last at University Hall, Gordon Square, W.C., Mrs. W. Blake Odgers presiding.

After the financial report had been read by the Hon. Treasurer, Mr. W. M. Blyth, Miss Marian Pritchard, one of the hon. secretaries, read the report. Having first given a message of regret from Mr. R. Hampson at his inability, through ill-health, to attend the meeting, she went on to show that last year 41 children had been received in the Home. Of these, 12 have remained more than a year, 16 stayed from three to six months, and the other 13 for a period averaging five weeks. Most of these came from different parts of London. The cases treated were: 19 "spinal and hip," 15 "debility," three "after mastoid operation," three "rickets," and one "infantile paralysis." Excellent results had been achieved in almost every instance. With regard to the financial position, the year's record could not be viewed from quite such a favourable standpoint. There had been a serious fall in the amount of subscriptions, due mainly, it is hoped, to some of these being late in coming in. For some years a generous unknown donor had sent £25, but this year it had not been received. The Committee could congratulate themselves and the subscribers on the thoroughness and true economy with which Miss Hope had continued to carry on the onerous task of lady superintendent during the last six years; a word of appreciation was also needed to be added to the three young nurses of the Home.

The President, in her address, bore fresh testimony to the excellent way in which the Home was managed, and alluded to the atmosphere of real home that prevailed in it. She thought that time spent there must result in a mental and moral uplifting which was not in the least valuable part of the healing. In conclusion, she moved the adoption of the report.

The Rev. W. Wooding, in seconding the motion, said that he considered that nothing could be more Christian than such an attempt to do this personal loving work for sick children.

The resolution was carried.

Mr. T. P. Young moved the re-appointment of officers and committee.

Miss Titford seconded the resolution, and it was carried.

Mrs. Thornhill then proposed thanks to the honorary medical officers and the Lady Superintendent and nurses This last motion was seconded by Mrs. Howard Young, and carried unanimously. The proceedings then terminated, after a few appreciative words of thanks from Miss Pritchard to Mrs. Odgers.

### NATIONAL SOCIETY FOR EMPLOYMENT OF EPILEPTICS.

THE annual meeting of Governors was held on Monday, June 20th. The chair was taken by Mr. E. Montefiore Micholls, and the reports of the Executive Committee and the honorary medical staff for the year 1903 were received and adopted. In the former report attention is called to the fact that the society is now prepared, as soon as funds are forthcoming, to undertake the care of young epileptic children. This had from the first been one of its principal objects; but, in consequence of its objections to certain conditions as to homes for epileptic children contained in the Elementary Education (Defective and Epileptic Children) Act, 1899, it was felt necessary to postpone undertaking this work until the clauses objected to were repealed, which has now at length been done by a measure promoted by this society and passed last session. It is anticipated by the society's medical advisers that the care of young children will eventually prove to be the most fruitful branch of the society's work, and the importance of commencing it at the earliest possible date is strongly urged. The report records various extensions made at the Chalfont colony during the year, including the erection of a home for 24 Hampshire cases and the commencement of an administrative building. The report of the Honorary Medical Staff states that, while there have been no special features of a novel kind from the medical aspect, the experiences of the year have fully confirmed their previous reports as to the success and usefulness of the colony system and methods in the care, treatment, and management of epileptics.

